# Effects of Oral Glucose Load on Endothelial Function and on Insulin and Glucose Fluctuations in Healthy Individuals

**DOI:** 10.1155/2008/672021

**Published:** 2008-03-04

**Authors:** A. Major-Pedersen, N. Ihlemann, T. S. Hermann, B. Christiansen, H. Dominguez, B. Kveiborg, D. B. Nielsen, O. L. Svendsen, L. Køber, C. Torp-Pedersen

**Affiliations:** ^1^Cardiology Research Clinic Y, Bispebjerg University Hospital, 2400 Copenhagen, NV, Denmark; ^2^Cardiology Department P and Laboratory, Gentofte University Hospital, 2900 Hellerup, Denmark; ^3^Internal Medicine Clinic I, Bispebjerg University Hospital, 2400 Copenhagen, NV, Denmark; ^4^Cardiology department, Rigshospitalet Univeristy Hospital, 2100 Copenhagen, Denmark

## Abstract

*Background/aims*. Postprandial hyperglycemia, an independent risk factor for cardiovascular disease, is accompanied by endothelial dysfunction. We studied the effect of oral glucose load on insulin and glucose fluctuations, and on postprandial endothelial function in healthy individuals in order to better understand and cope with the postprandial state in insulin resistant individuals. *Methods*. We assessed post-oral glucose load endothelial function (flow mediated dilation), plasma insulin, and blood glucose in 9 healthy subjects. *Results*. The largest increases in delta FMD values (fasting FMD value subtracted from postprandial FMD value) occurred at 3 hours after both glucose or placebo load, respectively: 4.80 ± 1.41 (*P* = .009) and 2.34 ± 1.47 (*P* = .15). Glucose and insulin concentrations achieved maximum peaks at one hour post-glucose load. *Conclusion*. Oral glucose load does not induce endothelial dysfunction in healthy individuals with mean insulin and glucose values of 5.6 mmol/L and 27.2 mmol/L, respectively, 2 hours after glucose load.

## 1. INTRODUCTION

Postprandial hyperglycemia is an independent risk factor for cardiovascular disease (CVD) in insulin-resistant individuals with type 2 diabetes or with the prediabetic state, impaired glucose tolerance (IGT) [1–10]. In line with these epidemiologic data, several studies have shown impairment of endothelial function, one of the earliest markers of atherosclerosis [11] after a meal or glucose challenge, in individuals with diabetes or IGT [12–17].

Endothelial function following acute hyperglycemia has also been studied in healthy individuals. Some groups have shown attenuation of endothelial function [18–26], whereas others have shown no change [27–31]. A few studies have shown acute hyperglycemia induced vasodilation in healthy individuals [32, 33], and only 2 studies (in vitro/ex vivo studies) have specifically reported glucose induced enhancement of the endothelial function [34, 35].

These seemingly conflicting results could be attributed to differences in methodology and subject characteristics: diverging periods of post-glucose load observations, varying periods of exposition to different concentrations of glucose, diverging criteria in subject selection, enabling versus blocking insulin’s physiologic response, and finally administrating oral glucose load versus intra-arterial or intravenous glucose administration.

Endothelial dysfunction is a predictor of cardiovascular risk; consequently, postprandial endothelial dysfunction might be a very early marker of atherosclerosis and, thus, an early potential target for the prevention of cardiovascular complications in insulin resistance. In order to better understand and cope with postprandial endothelial dysfunction and accompanying postprandial hyperinsulinemia and hyperglycemia in insulin resistant individuals, we find it important to study postprandial glucose and insulin fluctuations and postprandial endothelial function in healthy, insulin-sensitive people.

## 2. MATERIAL AND METHODS

### 2.1. Study population

Ten non-smoking subjects, with no history of cardiovascular or other chronic disease, with no signs of current acute disease, and no familiar disposition to glucose intolerance, were included after screening a total of 13 individuals. We assessed blood pressure, ECG, oral glucose tolerance test, calculations of body mass index (BMI) and homeostasis model assessment (HOMA), and biochemical parameters. Three subjects were excluded: two due to total cholesterol > 6 mmo/L and one due to history of anorexia nervosa. Participants' characteristics are shown on Table 1. All subjects gave written informed consent and the study was approved by the ethics committee for Copenhagen and Frederiksberg counties.

### 2.2. Study design

**Figure 1:**
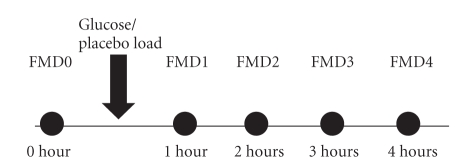
Flow chart showing the study sequence during a study day. FMD1, FMD2, FMD3, and FMD4, respectively. Flow mediated dilation study at 1, 2, 3, and 4 hours after glucose/placebo load. FMD0, flow mediated dilation study before glucose/placebo load.

Cross over study, *n* = 10. Subjects met on 2 separate days at 8:00 am after a 12-hour fast and after at least 18 hours abstinence from exercise. On the “glucose day”, endothelial function was measured before a 75 g oral glucose load (200 mL) and again at 1, 2, 3, and 4 hours after glucose load. On the “placebo day”, the same procedure was followed, but the 75 g glucose was substituted with placebo (200 mL tap water) as shown in Figure 1. The first FMD measurement of the day commenced between 8:30 and 9:00 am, glucose/H_2_0 was given between 9:00 and 9:30, and the last FMD measurement of the day ended between 13:30 and 14:00.

We extrapolated our post-glucose load findings to postprandial effects, since plasma glucose concentrations measured 2 hours after the ingestion of 75 g glucose have been found to be closely related to those measured after a standardized mixed meal [36].

### 2.3. Measurements of endothelial function

We used the **f**low **m**ediated **d**ilation (FMD) method [37, 38]. 20 minutes after supine rest, a pneumatic cuff was placed distal to the antecubital fossa, a 7.5 MHz linear array transducer (Cypress/Siemens, Siemens Medical Solutions USA, Inc. Mountain View, CA) was placed proximal to the antecubital fossa, and the brachial artery was viewed in a 2-dimensional (B-mode) longitudinal plane. A baseline picture was saved to ensure that the same brachial picture was found in the successive studies. After acquiring a two-minute baseline image, the cuff was rapidly inflated to 300 mmHg (VBM tourniquets, Braun Scandinavia) for 5 minutes, followed by rapid cuff deflation. Images were registered during 4 minutes after cuff deflation. Brachial diameters (at baseline and after cuff-deflation) were measured simultaneously by automated software (VIA online flow mediated dilation software, MD Medic) [39]. This software enabled us to precisely localize the greatest EDV since dilation was depicted graphically (as well as numerically).

The following formula was used to calculate relative change in FMD (expressed in %): ((Peak post cuff-deflation diameter − basline diameter)/baseline diameter) × 100 [40].

An intravenous cannula was inserted into a large antecubital vein in the left arm for blood sampling.

### 2.4. Biochemical measurements

Blood samples were drawn on both days before oral glucose load/placebo administration and at 15, 30, 60, 120, 180, and 240 minutes after plasma was separated after complete clot formation, subsequent to centrifugation, and thereafter frozen at −80°C for posterior analysis of insulin (Immulite 2000, DPC Scandinavia, Denmark).

Whole venous blood glucose was measured at the aforementioned times with HemoCue glucose (HemoCue AB, Angelholm, Sweden).

### 2.5. Data analysis

Data are presented as mean ± SEM. Comparisons between time points on the same curve (pre-oral glucose load, 1hPG, 2hPG, 3hPG, and 4hPG) for FMD, insulin, and glucose were evaluated with paired student *t* test for each curve separately. The level of statistical significance used was *P* < .05. For comparison of FMD time response studies between the 2 curves (post-glucose curve versus post-placebo curve), we used MIXED MODEL analysis (SAS statistical software version 9.1, SAS Institute, Cary, NC, USA).

Model assumptions of homogeneity of variance and normal distribution of residuals were checked graphically. Subjects entered the model as random effect as did the interaction between subject and time. Time and experimental protocols (glucose versus placebo) were entered as fixed effects.

## 3. RESULTS

### 3.1. Endothelial function

Delta FMD values (fasting FMD value subtracted from postprandial FMD value) ± SEM for post-glucose load and post-placebo load showed increasing FMD delta values for both days (see Table 2). The largest post-glucose load increase occurred at 3hPG (*P* = .009); compared to baseline, the increase was already significant at 1hPG (*P* = 0.03). The largest post-placebo increase also occurred at 3 hours post-placebo (*P* = .15) but was not significant (see Table 2). When we compared the two curves employing mixed models (post-glucose load curve versus post-placebo load curve), we found the curves to be statistically different (*P* = .0007).

### 3.2. Metabolic parameters

#### 3.2.1. Glucose

Post-glucose load mean blood glucose concentration values showed a significant increase at both 1hPG (*P* = .03) and 2hPG (*P* = .002). Conversely, a significant fall in blood sugar was observed at 4hPG (*P* = .002, *n* = 6 since the study was interrupted before the fourth postprandial hour in 3 subjects due to symptomatic hypoglycemia). As expected, post-placebo load values showed no significant change in blood glucose concentrations in the fasting state (see Table 2).

#### 3.2.2. Insulin

Mean insulin concentration post-glucose load values showed a marked increase at 1hPG (*P* = .0006) and fell rapidly thereafter, achieving pre-glucose-like values by 3hPG. Post-placebo load values fell across the 4 hour post-load period, which was already apparent at one hour post placebo load (*P* = .0006) (Table 2).

## 4. DISCUSSION

Our principal finding was that oral glucose load did not impair endothelial function in conduit arteries in our group of healthy, insulin-sensitive individuals with mean 2hPG of 5.58 mmol/L. We found maximum glucose and insulin peak concentrations at 1hPG. Furthermore our findings imply that oral glucose load might enhance endothelial function in these individuals.

Many studies have examined the effect of acute hyperglycemia on the healthy endothelium [18, 20, 22, 23, 30, 41]. These studies, however, cannot be directly compared to ours since both the methodology and the purpose of the studies diverge from our study. The aforementioned studies demonstrated that hyperglycemia induced impaired endothelial function, in healthy individuals, by either applying a local glucose/dextrose clamp or by intra-arterial glucose infusion, hence obtaining elevated hyperglycemic values (7.0 to 16.7 mmol/L). Yet, postprandial hormonal mechanisms, such as those leading to the “incretin effect” [42], may not be triggered when examined through the hyperglycemic clamp technique or local hyperglycemia. Some of these studies [30, 41] had also administered systemic ocreotide to suppress insulin secretion response to hyperglycemia. We did not find it appropriate to inhibit insulin secretion in order to study the physiologic endothelial post-glucose load response in healthy individuals since we suspected that the post-oral-glucose load insulin response might be pivotal in contributing to endothelial dependent vasodilation. Aside from keeping all hormonal responses intact, we chose to investigate post-glucose load endothelial function up to 4 hours after glucose load, because we were aware of the possibility that the endothelial response might not necessarily occur simultaneously with the increasing glucose and insulin concentrations. Our study is further strengthened by the fact that “time to peak” (the time it takes to achieve maximal dilation after cuff-deflation) varies individually, and that, by using our automated edge-detecting software, we have been sure of appreciating the largest dilation at each FMD measurement. Earlier studies using the FMD method have been forced to find a spot more or less blindly, between 40 and 90 seconds post cuff-deflation, for their measurements [19, 25].

As mentioned, differences in subject selection, particularly regarding postprandial blood glucose concentrations, might too have contributed to the apparently diverse findings reported in our study and other studies. A continuous relationship between increasing fasting and postprandial glucose concentrations and the risk of cardiovascular mortality in healthy, normoglycemic subjects has been documented [9, 43, 44]. Furthermore, a strong inverse relationship has been demonstrated between glucose levels, and endothelial function and intima media thickness (IMT) across the normoglycemic continuum; this was marked already at the 5.2 mmol/cut-off, and was even more significant with fasting glucose above 5.7 mmol/L [45]. Interestingly, a directly proportional risk gradient between CVD and postprandial glucose has been evidenced at1hPG [9].

In accordance with our selection criteria, our subjects were metabolic healthier, with lower mean fasting and postprandial glucose values than those in studies which employed a methodology similar to ours and, yet, demonstrated impaired endothelial function [21, 25, 26]. To illustrate, Title’s study [19] reported impaired endothelial function after oral glucose loading in 10 study subjects with a mean 1hPG of 7.9 mmol/L (as opposed to 6.2 mmol/L in our subjects). Thereby, the apparently contradictory results between Title's study and ours are actually complementary.

A limitation to our study is that it does not shed light on the cellular mechanisms responsible for the postprandial endothelial response. Yet, several mechanistic studies support our findings. Taubert’s group [34], for example, demonstrated that insulin's NO vasodilatory effect was augmented in the presence of relative hyperglycemia [34]. Indeed, in our subjects, there was a short and modest postprandial plasma glucose spike, followed by a larger postprandial hyperinsulinemic spike, which, in conjunction, led to an enhancement of the endothelial function when compared to baseline endothelial function. Conversely, our group [46] has previously proposed that persistent hyperglycemia inhibits PI3K-dependent signaling. Taubert's group reported that glucose and insulin stimulated NO release was impaired in the presence of hyperglycemia lasting beyond 2 hours, and that acute hyperglycemia did not increase NO release after prolonged exposure to hyperglycemia, leading them to suggest that enhanced superoxide generation in chronic hyperglycemia could be responsible for accelerated NO degradation. These findings could explain why studies where hyperglycemic clamps were employed, and/or examined subjects with relatively high postprandial values, have not found enhanced endothelial function [18–20, 23, 30, 47].

The greatest increment in endothelial function in our study occurred at 3 hours post-glucose load when glucose concentrations were back to baseline levels yet with insulin concentrations still 6 times higher than baseline insulin concentrations. Furthermore we observed a positive post-glucose load delta value at the cessation of the study (4hPG) for FMD and insulin concentrations. Interestingly, Cardillo et al. [48] found maximal insulin dilator effect after 2 hours of insulin infusion, leading them to postulate that insulin has a relatively slow-onset vasodilator effect. Accordingly, four hours of sustained hyperinsulinemia in the healthy has been shown to induce endothelial dependent vasodilation [49]. These observations could therefore explain our positive findings and why research groups that have limited their postprandial endothelial studies to 2 hours or less after a meal/glucose load [24] have not observed insulin induced endothelium dependent vasodilation following a hyperglycemic spike.

As aforementioned, post-glucose load glucose concentrations correlate closely to post-standardized meal glucose concentrations; yet we cannot directly compare our results with studies that have used standardized meals since these include lipids. Admitting that a standardized meal would have reflected a truer postprandial response, a standardized meal would have obscured the action of glucose alone on the endotheliuem, since it has been demonstrated that postprandial hyperlipidemia induces postprandial endothelial dysfunction in the healthy endothelium [50–52].

## 5. CONCLUSION

In short, we find that endothelial dysfunction is not a normal, physiologic, post-glucose load response. Instead, this study finds preserved post-glucose load endothelial function, thus implying that sugar derived from meals does not promote endothelial dysfunction, providing that postprandial insulin and glucose responses are preserved. It seems that the interaction of glucose and insulin on the endothelial function is affected both by the magnitude of glucose and insulin concentrations, and by the duration of exposition to these.

The immediate question arising from this study is whether by targeting both postprandial glucose and insulin peaks in insulin resistant individuals, so that both postprandial peaks resemble those of insulin sensitive individuals, enhances postprandial endothelial function. There is a pressing need for a greater therapeutic focus on the postprandial state, accompanied by a redefinition of the “normal” postprandial state, marking the true threshold for the onset of cardiovascular risk.

## Figures and Tables

**Table 1:** Subject characteristics. Data are presented as mean value ± SEM.

Variable	(*n* = 10)
Age (years)	41.1 ± 3.0
Gender (F/M)	4/6
Bo dy mass index (kg/m^2^)	23.2 ± 0.71
Waist circumference (cm)	80.7 ± 2.9
Glucose-fasting (mmol/l)	4.12 ± 0.12
Glucose-2 hour post-glucose load (mmol/l)	5.58 ± 0.32
P-fasting insulin (*μ*U/ml)	2.96 ± 0.37
HbA_1c_(%)	5.06 ± 0.2
P-fasting triglycerides (mmol/l)	0.75 ± 0.1
P-CRP (mg/l)	1.14 ± 0.1
HOMA (kg/m^2^)	0.65 ± 0.09

**Table 2:** Effects of glucose load and placebo load on FMD, blood glucose and plasma insulin. Data are presented as mean value ± SEM.

Times	Delta FMD (%)		Blood Glucose (mmol/l)		Plasma Insulin (*μ*U/l)	
	Glucose load	Placebo load	Glucose load	Placebo load	Glucose load	Placebo load
Pre-load	0	0	4.12 ± 0.12	4.46 ± 0.17	2.96 ± 0.37	4.24 ± 0.67
1 h	2.10 ± 0.84	0.19 ± 0.70	6.21 ± 0.79	4.54 ± 0.15	34.42 ± 4.99	3.30 ± 0.55
2 h	0.65 ± 0.85	1.66 ± 0.84	5.58 ± 0.32	4.64 ± 0.19	27.24 ± 3.03	2.89 ± 0.48
3 h	4.80 ± 1.4	2.34 ± 1.47	4.23 ± 0.37	4.53 ± 0.17	12.67 ± 2.54	3.44 ± 0.82
4 h	2.0 ± 1.39	0.9 ± 1.44	3.35 ± 0.17	4.27 ± 0.18	4.81 ± 0.88	2.90 ± 0.58

## ABBREVIATIONS

1hPG, 2hPG: 1-hour and 2-hour glucose values after an oral 75 glucose load
3hPG, 4hPG: 3-hour and 4-hour glucose values after an oral 75 glucose load
CVD: Cardiovascular disease
FMD: Flow mediated dilation
HOMA: Homeostasis model assessment
IGT: Impaired glucose tolerance
NO: Nitric oxide
PI3K: Phsophatidylnositol 3′-OH kinase
